# Implementation of Biological Control to the Integrated Control of Strongyle Infection among Wild Captive Equids in a Zoological Park

**DOI:** 10.1155/2018/4267683

**Published:** 2018-06-07

**Authors:** A. M. Palomero, J. A. Hernández, C. F. Cazapal-Monteiro, Fabián Arroyo Balán, M. I. Silva, Adolfo Paz-Silva, R. Sánchez-Andrade, María Sol Arias Vázquez

**Affiliations:** Control of Parasites Group (COPAR, GI-2120), Department of Animal Pathology, Faculty of Veterinary, University of Santiago de Compostela, 27002-Lugo, Spain

## Abstract

The integrated control of strongyles was assayed for a period of three years in wild equids (zebras, European donkeys, and African wild asses) captive in a zoo and infected by strongyles. During three years control of parasites consisted of deworming with ivermectin + praziquantel; equids also received every two days commercial nutritional pellets containing a blend of 10^4^ - 10^5^ spores of the fungi* Mucor circinelloides* +* Duddingtonia flagrans* per kg meal. Coprological analyses were done monthly to establish the counts of eggs of strongyles per gram of feces (EPG). The reductions in the fecal egg counts (FECR) and in the positive horses (PHR) were calculated fifteen days after deworming; the egg reappearance period (ERP) and the time elapsed from the previous deworming (TPD) were also recorded. Four anthelmintic treatments were administered during the assay, three times throughout the first 2 yrs, and another treatment during the last one. FECR values of 96-100% and 75-100% for the PHR were recorded. The ERP oscillated between eight and twenty-eight weeks, and the TPD ranged from four to eighteen months, increasing to the end of the trial. No side effects were observed in any of the equids. It is concluded that integrated control of strongyles among equids captive in a zoo can be developed by anthelmintic deworming together with the administration of pellets manufactured with spores of parasiticide fungi every two days.

## 1. Introduction

Wild animals captive in zoological parks are frequently confined to the same area, which enhances that elevated concentrations of parasites are attained in the ground [1]. This problem worsens due to stress, living conditions, and/or limitations of space increasing susceptibility of animals to some parasitic infections [2, 3].

Prior investigations suggested that parasites involving an intermediate host, as trematodes or cestodes, are more difficult to develop in zoos [4]; then those having a direct life-cycle are highly prevalent in captivity, especially parasites capable of surviving for long periods in the environment as strongyles or ascarids [5–7]. Clinical signs attributable to parasites are seldom observed in captive animals, which might point that subclinical infections develop in most of cases [8]. However, helminths can cause fatal infections if control fails [9, 10].

Control of parasites in zoo animals remains a pendant question, comprising the administration of parasiticide drugs [11, 12]. Nevertheless, analyses for establishing the presence of parasitic infection are rarely asked before the deworming, and the evaluation of the successful is seldom considered. Another inconvenience relies on that confinement of wild animals in the same parcel enhances the risk of exposure to infective stages of several parasites; thus frequent deworming can be needed, in a similar way to that reported in domestic animal species [13].

Recent investigations pointed out that certain saprophytic fungi are capable of performing antagonistic activity against parasitic stages (eggs, larvae) in the ground. Hyphae of* Pochonia chlamydosporia*,* Verticillium chlamydosporium*, or* Paecilomyces lilacinus* attach to the eggshells of trematodes, ascarids, and trichurids and penetrate and destroy the embryo inside [14, 15].* Duddingtonia flagrans* and* Monacrosporium thaumasium* elaborate traps in their mycelia where larvae of strongyles are immobilized and finally disrupted [16, 17]. A beneficial effect has been reported on avoiding infection by strongyles among wild captive equids, consisting of mixing chlamydospores of* D. flagrans* with feedstuff [13]. Herein, the integrated control of strongyles among equids from a zoological park was carried out; accordingly, zebras, African asses, and European donkeys were dewormed and fed pellets containing a blend of spores of* Mucor circinelloides* +* D. flagrans*.

## 2. Material and Methods

### 2.1. Zoological Park

The current trial was conducted in the Marcelle Natureza Zoological Park (Outeiro de Rei, Lugo, NW Spain; 43°4′14.71′′ N, 7°37′53.50′′ W), where wild animals are kept in an area of approximately 20-ha.

The zoo is opened to visitors from March to November. Most species are housed in fenced green parcels where they can exercise, enjoy the environment, and interact. Feces are removed daily from the parcels, prior to the opening hours, to keep them clean for the visitors [15].

Herbivorous graze in green fenced parcels and receive concentrate (pellets) every two days and water is available ad libitum. Hay is provided from December to February, when the pasture is scarce.

### 2.2. Equids

Three equid species living in the zoological park were used in the current trial. Two adult (>5 yr)* Equus quagga* (plains zebra) pasturing a 4038-m^2^ grassland, five adult* E. asinus* (European donkey) grazing a 2015-m^2^ meadow, and four adult* E. africanus asinus* (African wild ass) maintained in a sandy area of 2015-m^2^, which are sometimes provided forage cut from zones inside and outside of the park.

Deworming is the only measure, and four applications per year are at least administered. Since 2011, the Group for the Control of Parasites (COPAR; USC-2021 Research Group) is responsible for the monthly monitorization of the values of fecal egg-output of parasites, and consequently anthelmintic therapy is suggested if the numbers of strongyles per gram of feces (EPG) exceed 400 EPG.

### 2.3. Production of Fungal Spores for the Biological Control Approach

Spores of the ovicide fungus* Mucor circinelloides* (strain CECT 20824) and the larvicide fungus* Duddingtonia flagrans* (strain CECT 20823) were jointly obtained in the COPFr submerged culture medium [18–20].

### 2.4. Industrial Manufacturing of Pelleted Feed with Fungal Spores

Pelleted feed given to the equids was composed by forages, oil seeds and derivatives, cereal grains and byproducts, sugar cane processing byproducts, minerals, and amino acids (Table 1). The pellets were manufactured at Piensos Flores, S.L.U. (Lugo, NW Spain), by adding a dosage of 10^4^ - 10^5^ spores of* M. circinelloides* +* D. flagrans* per kilogram of milled ingredients. Prior to entering the pelletizer, the blend was injected steam at 75°C for circa 90 seconds and then the product was cooled, air-dried, and filled into 25 kg bags.

### 2.5. Experimental Design

The current trial was carried out between August 2014 and September 2017. Every two days, equids were given individually an approximate amount of 2.5 Kg of pellets containing spores of the parasiticide fungi.

Fecal samples were collected monthly and analyzed by the flotation test to determine the values of egg-output, expressed in numbers of eggs per gram of feces (EPG).

In August 2014, eggs of strongyles were observed in the feces of all equids (plain zebras, European donkeys, and African wild asses) by means of the flotation test, then a dosage of 1.07 g ivermectin + praziquantel (Equimax, Virbac, Spain)/100 kg body weight was administered.

As stated above, deworming was performed again when values of egg-output higher than 400 EPG were found.

### 2.6. Coprological Tests

By considering that rectal sampling of wild animals is not possible due to the difficulties to properly immobilize them to avoid being unnecessarily stressed, fresh fecal samples were taken directly from the ground of each parcel early in the morning, before keepers cleaned them. A total of four fecal samples were taken from zebras, ten from European donkeys, and eight from African wild assess, although it is possible that some of them could be represented via two samples. Feces were analyzed once in the lab through the flotation, sedimentation, and migration probes to determine the presence of parasites [20].

Efficacy of anthelmintic treatment was evaluated by taking fecal samples 15 days after the deworming, with the formulae [21]

(1) % Fecal egg count reductionFECR=1−FECpost-treatmentFECpre-treatment×100% PHR=1−number of positive horsespost-treatment⁡positive horsespre-treatment×100

The egg reappearance period (ERP) and the time lapsed from the previous deworming (TDP) were also estimated as follows:

ERP (egg reappearance period): the week after treatment when the FECR decreased below a cut-off value of 90% [22, 23].

TPD (Time from the Previous Deworming): number of months elapsed from the previous administration of anthelmintics.

### 2.7. Undesirable Effects

During the course of the trial, the occurrence of normal appetite was evaluated among all captive equids and if they ingest all the pelleted feed provided or refused it.

The activity of the digestive and respiratory systems and the skin integrity were tested in search of any disorder attributable to the ingestion of pellets with spores.

## 3. Results

At the beginning of the trial, eggs of strongyles were only observed in the feces of equids by means of the flotation test. Eggs of* Trichuris* sp. were occasionally identified throughout the assay. No coccidia, trematodes, cestodes, or lung worms were found.

### 3.1. Dynamics of Strongyles Fecal Egg-Output

**Figure 1:**
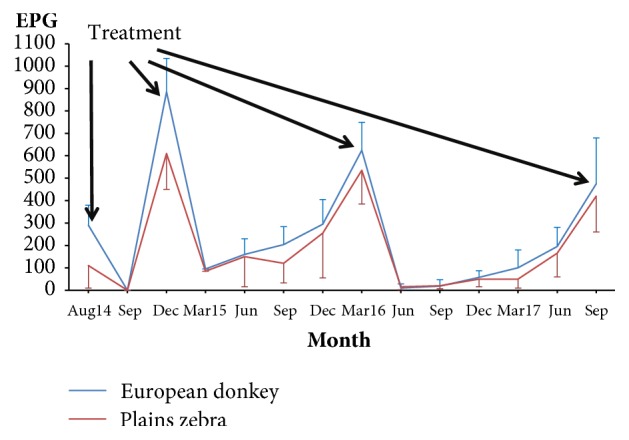
Kinetics of eggs of strongyles in the feces of wild equids feeding on grasslands in a zoological park. (EPG: eggs per gram of feces). Point values represent mean values and bars the standard error.

**Figure 2:**
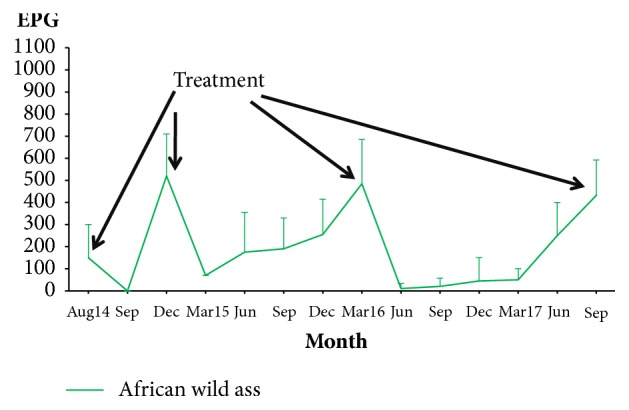
Kinetics of eggs of strongyles in the feces of wild equids maintained in a sandy area in a zoological park. (EPG: eggs per gram of feces).

Data regarding the kinetics of fecal egg-output are drawn in Figures 1 and 2. The EPG counts increased after the first administration of anthelmintics (August 2014) and peaked at December 2014, when EPG values higher than 400 were attained and thus all equids were treated again. The EPG counts decreased significantly, but a new increment from March 2015 to March 2016 was recorded, when numbers higher than 400 EPG were observed in the feces of all equids (Figures 1 and 2), thus deworming was administered again.

From April 2016 to August 2017, the strongyle EPG counts increased slowly and remained lower than 400 until September 2017, when new deworming was required.

As can be seen in Figures 1 and 2, the strongyle EPG numbers reduced throughout the trial. By considering the phases between deworming was applied (Table 2), the mean values of EPG reduced by 13-30% in the second period and by 34-48% in the third period.

### 3.2. Efficacy of the Strategy of Integrated Control

Fifteen days after the first administration of anthelmintics (August 2014), the FECR values were 100% for European donkeys, plains zebra, and African wild asses and the PHRs were 100% as well (none of the equids shed eggs in the feces) (Table 3). By considering the FECR values, an ERP of 8 weeks was recorded for zebras and European donkeys and 12 weeks for the African asses.

In December 2014, i.e., four months after the initial deworming, EPG counts exceeded the cut-off value (400 EPG) and anthelmintics were administered again, reaching full efficacy (FECR = 100%; PHR = 100%). The ERP was 12 weeks for zebras and European donkeys and 16 weeks for the African asses.

As summarized in Table 2, the next deworming (March 2016) happened 15 months later than the last treatment, with successful results. Only one individual from the European and the African donkeys remained passing eggs of strongyles by feces, and the ERP in all equids was 24 weeks.

Finally, anthelmintics were administered by September 2017 (i.e., 18 months after the last deworming), becoming fully effective. One of the European donkeys and one of the African asses were positive to the flotation test, and the ERP was 28 weeks in all captive equids.

### 3.3. Undesirable Effects

Refusal of ingestion of pellets did not occur at any time, and equids took all the feed provided, showing a normal appetite. No abnormal situations of constipation or diarrhea were observed.

Alterations in respiratory function as coughing, dyspnea or altered breathing patterns, fatigue, sneezing, itching, or nasal discharge were not recorded throughout the assay.

The visual examination of the skin confirmed the absence of rash, hair loss, flaking, eczemas, ulcers, reddened areas, blistering, or cracking.

## 4. Discussion

Certain changes introduced in the last decades in zoological parks were focused to improve the welfare and living conditions of captive animals. In this way, some policies and practices have been developed to ensure that animals living in captivity enjoy an appropriate environment, as keeping herbivores in parcels with grass. Under this situation, captive equids maintained in zoological parks are at risk of infection by parasites, and control strategies consist basically of deworming with drugs available for domestic horses. In the current trial, the effect of a strategy of integrated control of strongyles based on anthelmintic treatment (ivermectin + praziquantel) when the counts of eggs per gram of feces (EPG) exceeded 400 and feeding (every two days) commercial pellets industrially manufactured with spores belonging to the parasiticide fungi* Mucor circinelloides* (ovicidal activity) and* Duddingtonia flagrans* (larvicidal activity) was evaluated among wild equids (plain zebras, European donkeys, and African wild asses) captive in a zoological park. According to the World Association for the Advancement of Veterinary Parasitology guidelines [24], the effect of the deworming was measured fifteen days after the administration of anthelmintics and FECR (Fecal Egg Count Reduction) values of 100% were obtained. The finding of none of the equids passed eggs (PHR = 100%), together with a period of reappearance of eggs in feces (ERP) of eight weeks, confirmed the success of the therapy, in agreement with previous investigations [13, 25]. Due to EPG numbers which increased during a four-month period after treatment and counts higher than 400 (the initially cut-off point established) which were recorded, deworming was needed again. The next treatment was administered fifteen months later, becoming fully effective. Finally, all the equids were successfully dewormed eighteen months after the last treatment. When horses under continuous grazing were dewormed with ivermectin (one treatment) and fed daily pellets with spores of* M. circinelloides* and* D. flagrans*, no additional anthelmintic treatment was required for 64 weeks (16 months) [19].

Infection by strongyles takes place when forage contaminated with third-stage larvae (L3s) is ingested [26]. Eggs of strongyles shed in feces develop one first-stage larva inside, which exits off and moults into second- and third-stage larva, the infective phase [27]. Similarly, among horses kept under a continuous grazing regime, wild equids captive in a zoo can be exposed to high levels of infective larvae of strongyles.

Reduction of the risk of infection focuses often on the recurrent treatment of equids, with the objective to decrease the presence of eggs which might result in elevated counts of third-stage larvae in the ground [28]. However, certain anthelmintics might be ecotoxic on organisms in charge of enriching the soil, such as the dung beetles, and necessary for decomposing manure [29, 30]. Some measures have been pointed to limit the contamination of grasslands, as the regular manual collection of feces (every 2-4 days) or pasture rotation [27, 31]. In the zoo where the present investigation was carried out, feces are taken away daily, before the visitors come into the park, but captive equids are always housed in the same parcel because rotation is not possible.

With the aim of reducing the development and presence of L3 strongyles in the soil and so the administration of anthelmintics, the oral administration of chlamydospores of the nematode-trapping fungus* D. flagrans*, capable of trapping larvae of nematode in its mycelium, has provided successful results in lessening significantly the numbers of nematode infective stages in the ground [16, 32, 33]. In grazing horses receiving mycelium of* D. flagrans* antagonistic activity on cyathostomin larvae has been demonstrated in the fecal environment but also at a distance of around 20-40 cm from the fecal pats, explaining the reduction in the numbers of L3s in the soil [34]. The proper distribution of fungal spores can turn into a serious difficulty, especially among animal species captive in zoological parks due to the inconvenience to maintain a close contact with keepers. In a previous investigation carried out in the same zoo as the present trial, chlamydospores of* D. flagrans* were mixed with the pelleted feed previously to be given to captive wild equids. Satisfactory results in controlling strongyles were obtained, and counts lower than 400 EPG were recorded during a one-year period [13], but this strategy needs keepers to elaborate the premix every two days.

The demonstration of spores of* M. circinelloides* and* D. flagrans* which survived the industrial manufacturing of pelleted feed offers a very useful solution to their distribution and provides also a practical approach to implement strategies of integrated control [35, 36]. To our knowledge, this is the first investigation concerning the feeding of captive animals with pellets containing spores of parasiticide fungi. Due to the practical impossibility of maintaining separate control groups for each equid species, we have no basis on which to directly compare the efficacy of a program involving only anthelmintic treatment to that of our program integrating anthelmintic treatment and feeding pellets with fungal spores. However, there are available data confirming the utility of this strategy among domestic horses under a similar regime (continuous pasturing) [19]. It should be underlined that after a three-year period ingesting pellets with fungal spores, wild equids in the current trial did not show any undesirable effect concerning digestive and respiratory systems or the skin, in agreement with precedent investigations conducted on horses under a program of integrated control of strongyles based on deworming plus the administration of fungal spores [19]. In a recent assay involving two groups of lambs infected by* Haemonchus contortus*, a similar feed conversion was obtained by taking nutritional pellets with or without chlamydospores of* D. flagrans* and both diets provided similar growth rates and values of Packed Cell Volume, Body Condition Score, and* H. contortus* EPG [37]; significantly lower counts of L3s were obtained in the feces of lambs taking pellets with spores.

Finally, in the current trial, counts of eggs of strongyles were higher in zebras and European donkeys than in African wild asses, possibly due to differences in the paddocks where they are maintained. Zebras and European donkeys are living in two grasslands with abundant forage, while African asses do it in a sandy parcel, where forage is rarely observed (at the corners). Hence, it could be supposed that these equids would hardly become infected. Nevertheless, it is necessary to consider that grass cut in areas inside and outside the zoo is frequently provided, without any measure of control of possible contamination by L3s.

## 5. Conclusions

Commercial manufacturing of pelleted feed with spores of the filamentous fungi* M. circinelloides* and* D. flagrans* affords a very useful tool to their administration to wild equids captive at a zoological park. This procedure provides a novel and sustainable strategy for developing programs of integrated control of strongyles involving anthelmintic deworming also.

## Figures and Tables

**Table 1:** Nutrient composition of commercial pelleted feed given to wild equids captive in a zoological park.

Ingredients	Concentration
Crude protein	14%
Crude fat	2.9%
Crude fiber	12.5%
Calcium	1.5%
Phosphorous	0.65%
Sodium	0.53%
Magnesium	0.54%
Vitamin A	10000 UI/kg
Vitamin D3	1500 UI/kg
Vitamin E	42 UI/kg

**Table 2:** EPG mean values in the periods between deworming were applied to wild equids captive in a zoological park.

Period		Plains zebras (*n* = 2)	European donkeys (*n* = 5)	African wild asses (*n* = 4)
1^st^: September 2014 – December 2014	EPG	240 ± 325	392 ± 451	235 ± 268

2^nd^: January 2015 – March 2016	EPG Reduction	209 ± 182 13%	276 ± 208 30%	203 ± 155 14%

3^rd^: April 2016 – September 2017	EPG Reduction	120 ± 157 43%	142 ± 176 48%	135 ± 171 34%

EPG: eggs per gram of feces.

**Table 3:** Evaluation of a strategy of integrated control of strongyles affecting wild equids captive in a zoological park.

		Plains zebras (*n* = 2)	European donkeys (*n* = 5)	African wild asses (*n* = 4)
August 2014	FECR	100%	100%	100%
	PHR	100%	100%	100%
	ERP	8 weeks	8 weeks	12 weeks

December 2014	FECR	100%	100%	100%
	PHR	100%	100%	100%
	ERP	12 weeks	12 weeks	16 weeks
	TPD	4 months	4 months	4 months

March 2016	FECR	100%	97%	96%
	PHR	100%	80%	75%
	ERP	24 weeks	24 weeks	24 weeks
	TPD	15 months	15 months	15 months

September 2017	FECR	100%	96%	98%
	PHR	100%	80%	75%
	ERP	28 weeks	28 weeks	28 weeks
	TPD	18 months	18 months	18 months

FECR: fecal egg count reduction.

PHR: positive horses reduction.

ERP: egg reappearance period.

TPD: time from the previous deworming.
